# Coordination of spike timing among the neurons of the cerebellum

**DOI:** 10.21203/rs.3.rs-8681449/v1

**Published:** 2026-01-30

**Authors:** Mohammad Amin Fakharian, Elijah A. Taeckens, Alexander N. Vasserman, Alden M. Shoup, Reza Shadmehr

**Affiliations:** Laboratory for Computational Motor Control, Department of Biomedical Engineering, Johns Hopkins School of Medicine, Baltimore MD

## Abstract

We tend to think of neurons as either excitatory or inhibitory, but certain neurons chemically inhibit their downstream targets while electrically exciting their neighbors. For example, in the cerebellum, molecular layer interneurons (MLIs) inhibit Purkinje cells (P-cells) via release of GABA but promote spiking in each other via gap junctions. What is gained by having an inhibitory neuron excite its neighbor? Here, we recorded activities of P-cells and MLIs as marmosets performed saccadic eye movements and found that spike timing in pairs of neighboring neurons of the same type exhibited a mathematical regularity: as firing rates increased, rate of spikes that were within 1ms of each other grew disproportionately while 2–4ms intervals were suppressed. To uncover the purpose of this coordination, during saccades we recorded thousands of neuron triplets in which two MLIs converged onto a single target P-cell. When the MLIs spiked within 1ms of each other, they produced superposition of their individual effects on their target; a deep inhibition followed by a post-inhibitory rebound. However, when the MLIs spiked 2–4ms apart, the two spikes interfered with each other, producing partial cancellation. Thus, electrical coupling between inhibitory neurons orchestrated their spike timing so that as firing rates increased, the temporal intervals that induced constructive superposition were promoted while the intervals that caused destructive competition were suppressed.

## Introduction

Neurons are classified as excitatory or inhibitory based on how they influence their postsynaptic targets. However, in many regions of the brain, including the cerebral cortex^1^, thalamus^2^, and the cerebellum^3,4^, inhibitory neurons excite each other via electrical coupling. What role does electrical coupling among inhibitory neurons play during control of behavior?

In the cerebellum, two classes of inhibitory neurons, Purkinje cells (P-cells) and molecular layer interneurons type 1 (MLI1s), employ ephaptic coupling and gap junctions^4–6^ to promote spiking in their neighboring neurons of the same type, usually at a distance of 30um or less^5^. Here, we recorded from hundreds of P-cells and putative MLI1s (pMLI1s) in small neighborhoods (termed “cliques”) where cell pairs of the same type excited each other^7^. We discovered that during saccades, the timing of spikes exhibited a mathematical regularity: as the firing rates of individual neurons increased, the rate of spikes that were no more than 1ms apart grew disproportionately with respect to chance, while the rate of spikes that had a slightly longer temporal distance (2–4ms) remained at or below chance. Thus, a specific spike interval was promoted within each cell pair while a slightly longer interval was suppressed.

To understand the purpose of this proclivity, we focused on triplets of cells in which a pair of pMLI1s converged onto a single target P-cell. We recorded from thousands of such triplets and measured the average effect of each pMLI1 spike on its target. When the spikes in the upstream pair were within 1ms of each other, they induced a deep suppression in the probability of spiking in the target, then a post-inhibitory rebound. However, when the spikes were 2–4ms apart, they interfered with each other, producing partial cancellation. As a result, electrical coupling promoted spike intervals that produced superposition in the downstream target, while simultaneously reducing the likelihood of spike intervals that would produce interference. This made each P-cell a detector of coincident spikes in its upstream pMLI1s^8^.

These results suggest a rationale for why electrical coupling may be necessary for inhibitory neurons: because inhibition is followed by post-inhibitory rebound in the target neuron, the electrical coupling promotes production of spike intervals that result in constructive superposition on the downstream target, while suppressing the intervals that induce mutual cancellation.

Next, we noticed that unlike the pMLI1s, individual P-cells exhibited an unusual regularity in the intervals between their spikes. Whereas both cell types employed electrical coupling, only in P-cells spike timing remained predictable across a wide range of firing rates^9–12^. This regularity implied that each P-cell relied on an internal clock to generate its spikes. We wondered whether regularity of spike timing had any effects on coordinating spike timing among pairs of P-cells. The data revealed that when a P-cell produced a spike that ephaptically engaged another P-cell, that spike reset the internal clock that governed the spike timing of the neighbor. Thus, a single spike in one P-cell altered the timing of several subsequent spikes in neighboring cells. Simulations demonstrated that the clock-like regularity in the individual P-cells, in combination with ephaptic coupling among pairs of P-cells, enhanced coordination of spike timing, promoting 1ms or less spike intervals while simultaneously reducing the likelihood of spikes at 1–2ms intervals.

These results indicate that clock-like spiking in individual cells enhances coordination of spike timing between cells, providing a mechanism for inhibitory neurons to transfer information without interference.

### Spike interactions organized neurons of the cerebellar cortex into small networks

**Figure 1.**
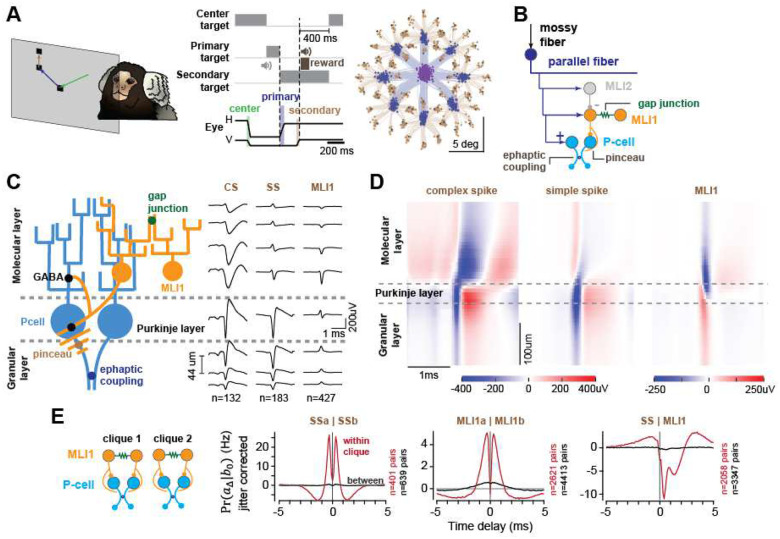
Characterization of sub-millisecond interactions among pMLIs and Purkinje cells in behaving marmosets. **(A)** Saccade task. **(B)** Network architecture, MLI2s inhibit MLI1s through GABAergic synapses, MLI1s inhibit P-cells via both GABAergic and electrical (pinceau) synapses; MLI1s are mutually connected through gap junctions; and P-cells co-activate each other through ephaptic coupling. **(C)** Schematic of synaptic organization and waveform alignment. Schematic illustrates relative arrangement and synaptic connections of P-cells and MLI1s based on rodent anatomical studies. Spatially aligned average waveforms from our marmoset recordings are overlaid at each corresponding layer. **(D)** Waveform alignment and layer identification. Average spatially aligned waveforms of complex spikes (left), simple spikes (middle), and MLI1 (right), obtained by identifying the Purkinje layer through the spatial boundary separating dendritic and axonal components of complex spike waveforms within each clique (see methods). **(E)** Within and between clique jitter-corrected conditional probabilities showing the likelihood that a spike occurred in one neuron given a spike in another neuron, across time delays, for P-cells and MLI1s. Error bars are SEM.

We trained marmosets to make saccades to visual targets (Fig. 1A) and used Neuropixels and other silicon probes to record from 154 definitive P-cells (CS and SS) and 734 pMLI1 from lobules VI and VII of the vermis (Fig. S1). In an earlier report we described the average firing rates of neurons in this dataset^7^. Here, our focus is on spike timing.

We identified the P-cells based on their simple and complex spikes (SS and CS) and then used the distinct shapes of the CS waveforms in the dendritic tree and axon of each P-cell^13,14^ to identify the molecular, Purkinje, and granular layers (Fig. 1C, Fig. S2). In the molecular layer, we labeled neurons that inhibited the P-cells at 1ms latency or sooner as pMLI1s (pMLI1, Fig. 1E)^15–17^. The pMLI1s tended to exhibit a negative spike waveform in the molecular layer but a positive waveform in the granular layer, an identifying feature of a pinceau (Fig. 1D)^18^.

In the molecular layer we also identified pMLI2s based on their inhibitory interactions with pMLI1s (Fig. S3), and the fact that climbing fibers strongly and broadly excited the pMLI2s, but only weakly and briefly the pMLI1s (Figs. S3 and S4)^17,19–21^. However, our pMLI2 numbers were relatively small (Fig. S1, n=60) and thus our focus here is on pMLI1s and P-cells.

We next clustered the neurons into small neighborhoods based on their interactions^7^. An example is shown in Fig. S2A. The spatial span of this probe allowed us to record from neurons in four Purkinje and molecular layers. However, only some of the cells interacted with each other. To visualize these interactions, we began with the jitter corrected^22,23^ conditional probability of a cell producing a spike at time *t* + Δ, given that another cell produced a spike at time *t* (Fig. 1E). Using the result of this conditional probability at short-latency delay (using 1ms time bin, maximum absolute interaction with a delay of Δ ∈ {0,1,2,3} ms), we formed an adjacency matrix (Fig. S2A) where the numerical value in each element of the matrix was the strength of the spike interaction between the two cells^7^. Next, we applied graph spectral clustering to the values in the matrix and identified boundaries that divided the neurons into neighborhoods in which cells strongly interacted with one another^7^, i.e., cliques.

To visualize the interactions within and between cliques, we plotted the high-resolution (0.1ms bin) conditional probability of a neuron producing a spike at time *t* + Δ, given that another neuron produced a spike at time *t* (Fig. 1E). Using jitter-corrected results, we found that if two P-cells resided in the same clique, then an SS in one P-cell was followed by ~26 Hz increase in the firing rate of another P-cell at 0.5ms latency, an interaction consistent with ephaptic coupling^5^ (Fig. 1E, SSa|SSb, red trace). This interaction was absent when the two P-cells belonged to different cliques (Fig. 1E, black trace). If two pMLI1s were in the same clique, then a spike in one pMLI1 was followed by ~5 Hz increase in the firing rate of another pMLI1 at 0.5ms latency, an interaction consistent with gap junctions^17^, but not if the two pMLI1s were in separate cliques (Fig. 1E, MLIa|MLIb). If a P-cell and a pMLI1 belonged to the same clique, then the pMLI1 strongly inhibited the P-cell, producing a bimodal pattern of suppression that, on average, exhibited a strong initial inhibition in the SS rates at 0.4ms latency, followed by a second, weaker inhibition at 1.4ms latency (Fig. 1E, SS|MLI, also Fig. S5), reproducing the pinceau and GABA-induced inhibitions in slice preparations^18^.

The cliques also organized the climbing fibers (although these interactions were not included in the clique clustering process). A complex spike produced complete suppression of the simple spikes in the parent P-cell (Fig. S1A), but also briefly inhibited the neighboring P-cell (Fig. S4D), likely via ephaptic coupling^14^. This effect was present only if the P-cell and the climbing fiber were in the same clique. The complex spike also produced brief excitation of pMLI1s^21^, and broad excitation of pMLI2s (Fig. S4), likely via spillover^15,16^, but only if the climbing fiber, the pMLI1s, and the pMLI2s were in the same clique. The climbing fibers also exhibited synchrony, but unlike the P-cells and the pMLI1s, CS coordination was present both within and between cliques with wider temporal profiles (Fig. S4A).

In summary, spike interactions clustered the neurons into small networks, termed cliques^7^. Within a clique the P-cell pairs and the pMLI1 pairs exhibited sub-millisecond coordination of spike timing, while the pMLI1s inhibited the P-cells at sub-millisecond latencies.

### Spike timing in cell pairs displayed a mathematical regularity

**Figure 2.**
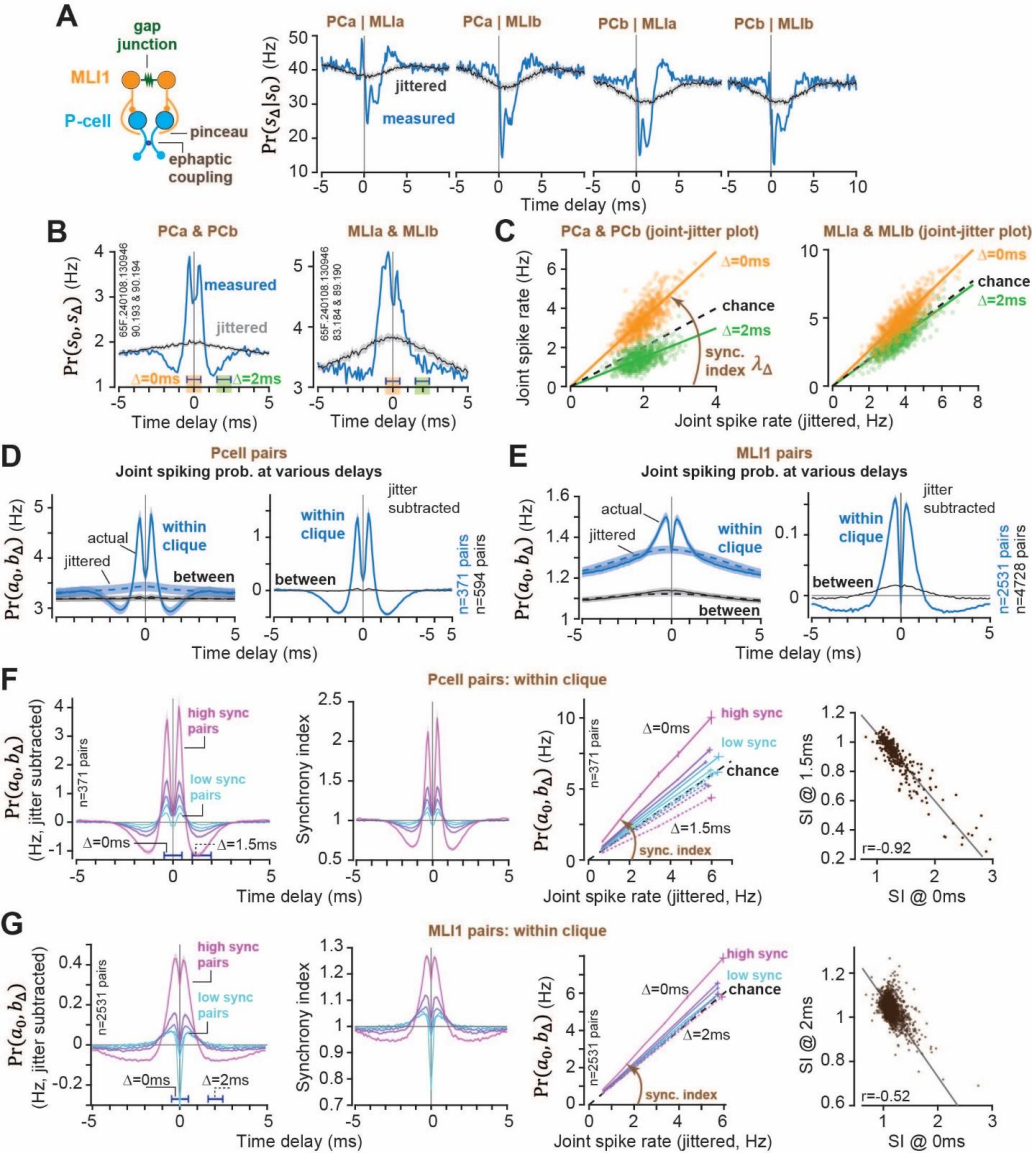
Synchrony increases linearly with firing rates in both pMLI1s and P-cells. Data from the entire recording. **(A)** Conditional probability of a P-cell given a pMLI1 spike at 0ms (blue), compared to the same probability computed after jittering pMLI1 spikes (black; jitter window = 5ms, n=10 jitter iterations), shown for a sample P-cell and pMLI1 pairs in the third clique in the Neuropixels recording in Figure 1E (measured from the whole recording). **(B)** Joint probability traces for the same P-cell pair (top) and pMLI1 pair (bottom), alongside their corresponding jittered controls (black). **(C)** Joint-jitter plots for the pairs in (B), each dot represents the number of synchronous spikes (within 1ms window; orange = 0ms, green = 1.5ms delay) versus the average number of synchronous jittered spikes (n=10 jitter iterations), calculated in non-overlapping 10s bins across the full recording. Lines indicate linear fit to the data points (only slope). **(D)** (left) Average raw (solid) and jittered (dashed) joint probabilities for all recorded P-cell pairs within (blue) and between (black) cliques. (Right) Jitter-corrected joint probabilities for the same groups. **(E)** Same as (D), but for all pMLI1 pairs. **(F)** From left to right: Jitter-subtracted cross-correlograms of P-cell pairs grouped by connectivity strength. Synchrony index. Joint-jitter plots at 0ms (solid) and 1.5ms (dashed) delays for each group. Synchrony indices at 0ms vs. 1.5ms delay (each dot is one pair). **(G)** Same analyses as in (F), but for pMLI1 pairs. Error bars are SEM.

A typical recording session lasted 3.02±0.3 hours (median±MAD, Fig. S1C), during which the neurons modulated their firing rates as the animal engaged in various behaviors, including eye movements^24^ and tongue movements^25^. To ask whether spike timing between cell pairs was patterned or merely random, we designed a new way to visualize the data, termed *joint-jitter plots* (Fig. 2C). To explain these plots, we begin with an example.

Consider a clique that included a pair of P-cells (PCa and PCb, Fig. 2A, Fig. S2D), and a pair of pMLI1s (MLIa and MLIb). To test whether the pMLI1 interacted with the P-cells, we computed the conditional probability that the P-cell produced a simple spike at time *t* + Δ, given that the pMLI1 produced a spike at time *t* (Fig. 2A). As a control, we jittered the spikes in the pMLI1 and recomputed the conditional probability (Fig. 2A, gray line). To establish confidence intervals, we repeated the jittering 10 times. The results revealed that each pMLI1 inhibited both P-cells, and this inhibition reached its peak at a latency of less than 0.5ms after the pMLI1 spike, a pattern consistent with ephaptic inhibition via pinceau^18^.

To measure coordination of spike timing between the P-cells, we measured the joint probability of spiking in a window of size *w* = 0.1ms and then shifted this window across time delays Δ (Fig. 2B, blue line). As a control, we jittered the spikes in one of the P-cells and recomputed the joint probability (Fig. 2B, gray line). The joint probability exceeded the jittered values at around ±0.4ms delay, then fell below those values at ±1.5ms delay (Fig. 2B, top row), a pattern consistent with ephaptic coupling between the two P-cells^5^.

To visualize how the joint spike rate varied as a function of firing rates, we generated a new spike train that placed a spike only when both P-cells spiked during a 1ms window centered at Δ = 0ms delay. As a control, we generated a spike train from the jittered data, then computed the rate of the joint spiking in 10s windows of time and plotted it against the rate from the jittered data at the same window of time. We named this the *joint-jitter plot* (Fig. 2C). The x-axis of the joint-jitter plot presents the rate of joint spikes that are expected by chance, i.e., the rate expected if the two neurons were independent of each other. The y-axis is the rate that is observed in the data. These plots revealed a mathematical pattern: the rate of joint spiking was approximately a linear function of the jittered rate. For example, at Δ = 0ms delay the joint rate exhibited a slope of greater than one (one implies chance), and at Δ = 2ms delay the joint rate had a slope of less than one.

A similar pattern was present in the two pMLI1s. The rate of joint spiking at Δ = 0ms grew linearly as a function of the rate of the jittered data and had a slope greater than one (Fig. 2C, bottom row). In contrast, at Δ = 2ms, the rate of synchronous spikes was at chance. Thus, the joint-jitter plots described a pattern in how the spikes of P-cell pairs, and MLI1 pairs, were tiled in time: the probability of neuron *b* spiking at time *t*, and neuron *a* spiking at time *t* + Δ, i.e., the joint spiking probability, was approximately a linear function of the jittered rates:

(1) $$\text{Pr}\left(a_{\Delta },b\right)=\lambda_{\Delta }\;\text{Pr}(a_{\Delta },b_{\text{jitt}})$$

The term *λ*_Δ_ is termed synchrony index and is the slope of each line in Fig. 2C. Eq. (1) implied that as the firing rates of the two neurons changed, the rate of spikes that were Δ ms apart from each other remained a constant multiple of the individual firing rates (because Pr(*a*, *b*_*jitt*_) ≈ Pr(*a*) Pr(*b*)). For example, as the firing rates of two P-cells increased during behavior, Eq. (1) implied that so did the number of spikes at Δ = 0ms latency with respect to chance, but with a slope that was invariant to the firing rates.

Eq. (1) is noteworthy because its predictions differed with that of a recent paper^26^. Herzfeld et al.^26^ suggested that as P-cell firing rates changed, the difference between joint and jittered rates, termed covariance, remained constant. In contrast, Eq. (1) states that as firing rates change, the ratio of the joint to the jittered probabilities remaine constant.

We tested the predictions of Eq. (1) by computing the joint probability of spikes at various delays across the entire recording for all P-cell pairs (Fig. 2D, left plot, solid blue line), and all pMLI1 pairs (Fig. 2E, left plot). In comparison to the jittered spikes, the joint spiking in P-cell pairs within a clique exhibited a sharp peak at Δ = ±0.5ms delay (Fig. 2D, jitter subtracted), then a minimum at Δ = ±1.5ms delay. However, between cliques the joint spiking was at chance. The pMLI1s exhibited a similar pattern of joint spiking within cliques, and a small but non-zero pattern of joint spiking between cliques (Fig. 2E, right).

Among the cells within a clique, some pairs exhibited much stronger joint spiking at Δ = 0ms than others. We divided the within clique pairs into five groups based on the strength of their jitter normalized joint probability (Fig. 2F, left subplot), then made joint-jitter plots for each group. The results revealed an orderly increase in the slope of the joint-jitter plots (Fig. 2G). Critically, the ratio of the joint probability of spiking at Δ = 0ms delay with respect to the jittered probability (Fig. 2F) was a near perfect predictor of the slope of the joint-jitter plot for each neuron pair (Fig. S9).

The jitter-corrected cross correlation for Δ = 1.5ms delay was negative (Fig. 2F, left subplot), suggesting that the joint spikes recorded at this delay were less frequent than expected by chance. Indeed, the joint-jitter plot confirmed that as firing rates increased, the rate of joint spikes at this delay fell below chance (Fig. 2F, middle subplot). Among the P-cells, the greater the synchrony index at Δ = 0ms delay, the smaller the synchrony index at Δ = 1.5ms delay (Fig. 2F, right subplot). Thus, as the P-cell firing rates increased, the rate of joint spikes at Δ = 0ms delay grew above chance while the rate of joint spikes at Δ = 1.5ms delay fell below chance. In other words, the simple spikes became better aligned with each other as firing rates increased.

Analysis of the pMLI1 pairs produced very similar results (Figs. 2G). Jitter-corrected joint probability stratified pMLI1 pairs based on the strength of their coupling, while the coupling strength defined the slope of the joint-jitter relationship. Like the P-cells, the greater the synchrony index at Δ = 0ms delay, the smaller the synchrony index at Δ = 2ms delay.

In summary, spike timing in the P-cells and pMLI1s exhibited a mathematical pattern: the probability that a pair of neurons would generate spikes at delay Δ from one another grew as a function of their jittered rates, with a slope that was greater than one for Δ = 0ms delay, but less one for slightly longer delays (slope of one implies chance). This means that as two neurons in a clique increased their firing rates, they promoted the spikes that were within 0 ± 0.5ms of each other, while suppressing (in case of P-cells) or maintaining at chance (in case of pMLI1s) the spikes that were within 1.5 ± 0.5ms of each other.

### Coordination of spike timing peaked near the onset of saccade deceleration

**Figure 3.**
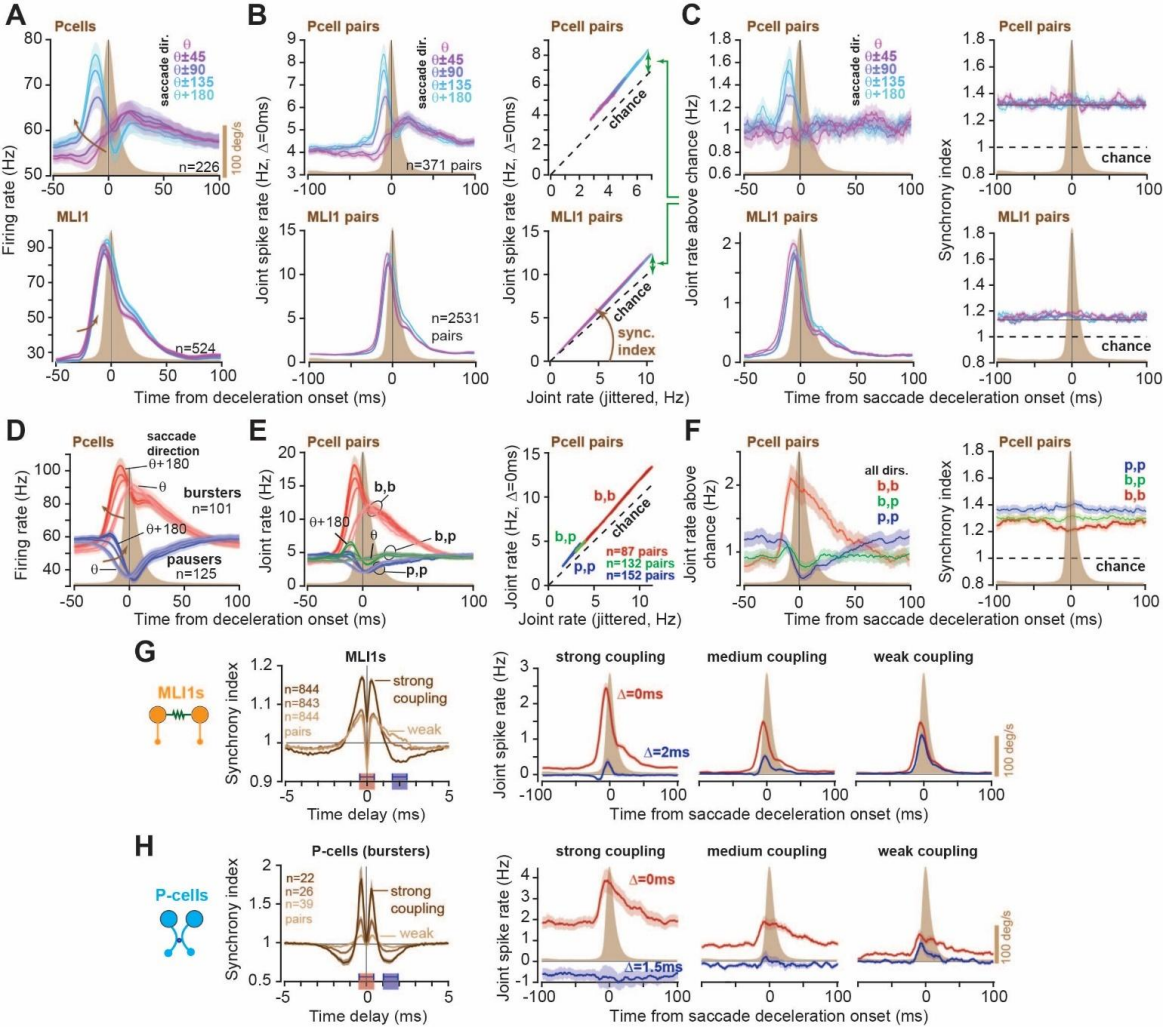
Synchronous spikes are promoted during saccades while asynchronous spikes are suppressed or remain at chance. Data from the saccade periods only. **(A)** P-cell (top) and pMLI1 (bottom) average firing rates aligned to saccade deceleration onset for different movement directions (aligned to each clique’s potent direction). **(B)** (Left) Same as (A) but showing the average joint spike rate at Δ = 0ms delay for each P-cell pair. (Right) Joint-jitter plots for all P-cell (top) and pMLI1 (bottom) pairs from −100 to 100ms relative to saccade deceleration onset, shown separately for each saccade direction (aligned to the clique’s potent direction). **(C)** Average jitter-subtracted (covariance; left) and synchrony index (right) for all P-cell (top) and pMLI1 (bottom) pairs within a clique, aligned to saccade deceleration onset. **(D-F)** Same as in (A-C) but separately for burster and pauser P-cell groups. **(G-H)** Grouping of pMLI1s and P-cells based on strength of electrical coupling. (Left) cross correlations represented as synchrony index, 1 is chance. (Right) Joint spike rate at various delays during saccades for groups of strong, medium, and weakly coupled pairs. Error bars are SEM.

Fig. 3A presents the firing rates of P-cells and pMLI1s for saccades in various directions. Here, *θ* is the saccade direction for which complex spike activity was maximum, and *θ* + *π* is the direction for which the complex spike activity was minimum^24,27^. The direction *θ* is important because it specifies the direction of the potent vector of the clique^7^. This means that when a P-cell is suppressed during a saccade, the eyes are displaced in direction *θ*^28^. The P-cells as a population showed an early increase in their firing rates for saccades in direction *θ* + *π*, and a late increase for saccades in direction *θ*. In contrast, the pMLI1s increased their rates for all saccades, displaying a slight shift in timing as a function of saccade direction.

As P-cell firing rates changed during saccades, so did the rate of spikes that were within 0 ± 0.5ms of each other (Fig. 3B, left column). However, regardless of saccade direction, the joint rate remained constrained to a line as a function of the jittered rates (Fig. 3B, right column). For example, during a saccade in direction *θ* + *π*, the firing rates increased then decreased, but the joint spike rate moved along a line without hysteresis (Fig. S6, Fig. 3B right column). During a saccade in direction *θ*, the firing rates were lower and delayed, but the joint spike rate was constrained to the same line (Fig. S6).

To compute the rate of synchronous spikes with respect to chance, we subtracted the jittered rate from the joint rate. Because the slope in the joint-jitter plot was greater than one, the rate of synchronous spikes above chance increased as the firing rates increased (Fig. 3C, left column). For the P-cells, the rate of synchronous spikes reached a peak before deceleration onset (Fig. 3C, left column), specifically for saccades in direction *θ* + *π*, i.e., the direction opposite to the potent vector. For the pMLI1s, the synchronous rate reached a peak just before deceleration for all saccade directions. Crucially, as predicted by Eq. (1), synchrony index *λ*_Δ_ remained roughly constant during all saccades for both P-cells and pMLI1s (Fig. 3C, right column).

Notably, during saccades in direction *θ* + *π*, the rate of synchronous spikes above chance was roughly 5 times greater if the P-cells were within a clique as compared to between cliques (Fig. 3C & S7A). This is because if the P-cells belonged to disparate cliques, there was still an increase in the rate of synchronous spikes during saccades (Fig. S7), but that increase remained near chance levels. Among the pMLI1s, the rate of synchronous spikes above chance was 2 times greater if the cell pair belonged to the same clique.

During a saccade some P-cells increased their rates while others decreased their rates, i.e., bursters and pausers (Fig. 3D). Thus, our cell pairs within a clique consisted of three groups: burster-burster (bb, n=87 pairs), burster-pauser (bp, n=132 pairs), and pauser-pauser (pp, n=152 pairs). These numbers implied that the two types of P-cells often coexisted in close spatial proximity, with a bias toward pauser-pauser pairs. As expected, the bb group showed a strong increase in the joint spike rate during saccades, the bp group showed little change, while the pp group exhibited a decrease (Fig. 3E, left subplot). However, regardless of whether the P-cells were bursters or pausers, the joint spike rate remained constrained to the same line (Fig. 3E, right subplot). That is, irrespective of the rate changes in P-cell pairs during saccades, the joint rate exhibited a linear relationship as predicted by Eq. (1) (Fig. S8).

Because saccades are only 30–40ms in duration, we were concerned that our ability to measure synchronous rates may be limited. To verify the robustness of our findings, we confirmed that for any pair of neurons, their synchrony index as computed during saccades was the same as the synchrony index computed during the entire recording (Fig. S9).

We next extended our analysis from synchronous spikes to spikes that were slightly asynchronous, i.e., 1.5–2ms apart. We stratified the neurons based on their strength of coupling (Fig. 3G & 3H) and found that in pMLI1 pairs with strong coupling, a rise in synchronous rates accompanied only small changes in asynchronous rates, but in pairs with weak coupling the synchronous and asynchronous rates matched each other. In P-cells with strong coupling, asynchronous rates were suppressed below chance during saccades, whereas in P-cells with weak coupling the two rates nearly matched.

Thus, for cell pairs within a clique, as the firing rates changed during a saccade, the rate of spikes within 0 ± 0.5ms of each other remained constrained to a single line above chance and reached a peak near deceleration onset. In the P-cell and the pMLI1 pairs that had strong electrical coupling, an increase in firing rates not only increased the rate of synchronous spikes, it also suppressed or kept at chance the rate of asynchronous spikes.

### Synchronous spikes produced superposition, but asynchronous spikes caused interference

Did production of synchronous spikes make a difference in the downstream target? Was production of asynchronous spikes somehow counterproductive? To answer these questions, we began by quantifying the effects of individual pMLI1s on the P-cells.

**Figure 4.**
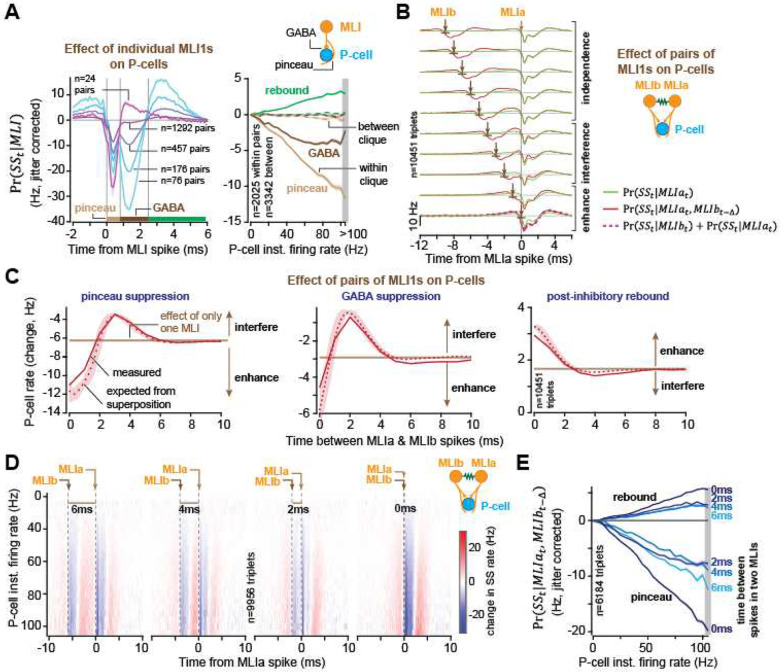
Synchronous pMLI1 spikes induce superposition on the target P-cell whereas asynchronous spikes induce interference. **(A)** (left) Conditional probability of P-cell simple spike given a pMLI1 spike at 0ms reveals three sequential phases corresponding to pinceau (electrical) suppression, GABAergic suppression, and post-inhibitory rebound. Pairs are binned by strength of GABA suppression, revealing varying contributions of pinceau and GABA effects across pairs. (Right) Average jitter-corrected conditional probabilities for each phase (light brown: pinceau, dark brown: GABA, green: rebound) as a function of the P-cell’s instantaneous firing rate, grouped by within-clique (solid) and between-clique (dashed) pairs. **(B)** Average effect of two pMLI1 spikes on a P-cell at different inter-spike intervals (each row; red lines). The green trace shows the effect of pMLI1a alone, and the dashed red trace shows the linear sum of individual effects from pMLI1a and pMLI1b. **(C)** Effects of spikes in pairs of pMLI1s on their target P-cell as a function of their temporal distance. Dotted line is the expected value if the effects of the two spikes summed linearly. The y-axis is the change in probability of spiking in the P-cell. The horizontal line is the expected effect on the P-cell if only one MLI had spiked. The solid red line is the measured effect, i.e., Pr(*SS*_*T*_|*a*_*t*_, *b*_*t*+Δ_). This is the probability that the P-cell spiked during the period *T*, given that MLIa spiked at time *t*, and MLIb spiked at time *t* + Δ. The dotted red line is the expected effect due to superposition, i.e., Pr(*SS*_*T*_|*a*_*t*_) + Pr(*SS*_*T*_|*a*_*t*_, *b*_*t*+Δ_). (left) The pinceau period, *T* = (*t* + 0) *to* (*t* + 0.8) ms period. (middle) The GABA period, *T* = (*t* + 0.8) *to* (*t* + 2.5) ms period. (right) The post-inhibitor rebound period, *T* = (*t* + 2.5) *to* (*t* + 6) ms period. **(D)** Same analysis as (B), but effects are binned by the P-cell’s instantaneous firing rate (from left to right: 6ms to 0ms intervals). **(E)** Pinceau suppression and rebound average amplitudes as a function of P-cell instantaneous firing rate for different delays between spikes in pairs of pMLI1s. Error bars are SEM.

For the cells that were in the same clique, we organized the data into pairs consisting of one pMLI1 and one P-cell (n=1403 pairs) and then computed the probability of simple spikes at time *t* + Δ, given that the pMLI1 produced a spike at time *t*. We then jitter corrected this probability and found that the inhibitory effect of the pMLI1s was bimodal in time: after the pMLI1 spike there was an initial period of P-cell inhibition that peaked at a latency of ~0.4ms, and then a second period of inhibition that peaked at a latency of ~1.4ms (Fig. 4A, left). The timing of the initial peak was consistent with electrical interaction at the pinceau, and the timing of the second peak was consistent with chemical interaction via GABA^18^. Some pMLI1s had a strong pinceau, some had a strong GABA, and some had both^29^. Notably, the P-cell inhibition was followed by a rebound that began at 2.5ms following the pMLI1 spike (Fig. 4A, left, green horizontal bar). The strength of pMLI1 interactions with P-cells was indistinguishable between P-cell bursters and pausers (Fig. S10).

Of course, by using conditional probabilities we were not directly measuring the suppressive effect of an MLI1 spike on a P-cell (inhibitory current to the P-cell), but rather the change in the P-cell’s probability of spiking. This measure depended on the P-cell’s overall firing rate, which reflected other excitatory and inhibitory inputs. For example, when the P-cell was firing slowly, the apparent effect of a single MLI1 spike would appear smaller than when the P-cell was firing rapidly. To account for the state-dependent effect of MLI1s on the P-cells, we adapted the 3D auto-correlogram approach^30^ and calculated the conditional probabilities as a function of the P-cell’s instantaneous firing-rate (Fig. 4A, right). We found that when the P-cell was bursting, the effect of the pMLI1 spike was greater, producing a larger initial inhibition, followed by a larger subsequent rebound.

Next, we considered triplets of neurons (n=10,451 triplets) composed of two pMLI1s, termed MLIa and MLIb, and one P-cell (Fig. 4B). We organized the data based on time Δ between two consecutive spikes: a spike in MLIb at time *t* − Δ followed by a spike in MLIa at time *t*, then measured the effect that each spike had on the simple spikes. For example, consider when the two spikes were more than 6ms apart (Fig. 4B, top row). The green line shows the result when only MLIa spiked, and the red line shows the result when both MLIs spiked. Each spike inhibited the P-cell, but their effects did not add because the events were too far apart, i.e., independence. As the two spikes occurred closer together, their combined effects began to sum so that when they occurred synchronously, the joint event roughly doubled the effect of a single event (Fig. 4C). However, when the two spikes were 2–4 ms apart, the result was interference, i.e., a smaller response that would have been produced if only one pMLI1 had spiked, because the inhibition from the latter spike overlapped with the rebound from the former. Thus, synchronous spikes were beneficial because they produced superposition whereas slightly asynchronous spikes were detrimental: because they produced interference.

Notably, the effect of the pMLI1 spikes depended on the state of the P-cell. To control for this, we computed the state of the P-cell via its instantaneous firing rate and found that for higher P-cell firing rates, the synchronous spikes in the pMLI1s exhibited a larger inhibition, followed by a larger rebound (Figs. 4D & 4E). For example, when P-cell firing rates were around 100 Hz, a rate attained by the bursters during saccades, a synchronous spike in a pair of MLIs doubled the probability of P-cell suppression, and also doubled the probability of a rebound simple spike, as compared to when the two MLI spikes were 4ms or more apart.

In summary, when the spikes in a pair of neurons were synchronous, their effect on the downstream target was superposition, but when the two spikes were only slightly asynchronous, the result was interference.

### Ephaptic coupling allowed neighboring P-cells to align their internal clocks with each other

Spike timing coordination occurs not just between two neurons, but also within a single neuron. For example, in individual P-cells (but not MLIs) spike timing is highly “regular”, meaning that spike timing is predictable^9–12^. This implies that as a first approximation, we can think of a P-cell as relying on an internal clock to generate its spikes. At any moment in time, this internal clock runs at a speed defined by the neuron’s instantaneous firing rate, and thus the passage of time from one spike to the next is the phase of the clock. This phase starts at zero when the cell fires, then reaches 2*π* at the time when we expect the cell to fire again. This analogy allows us to ask the following question: when a P-cell generates a spike that engages its neighboring P-cell via ephaptic coupling, does that spike reset the clock of its neighbor? In other words, is ephaptic coupling a mechanism with which P-cells coordinate their internal clocks with each other?

**Figure 5.**
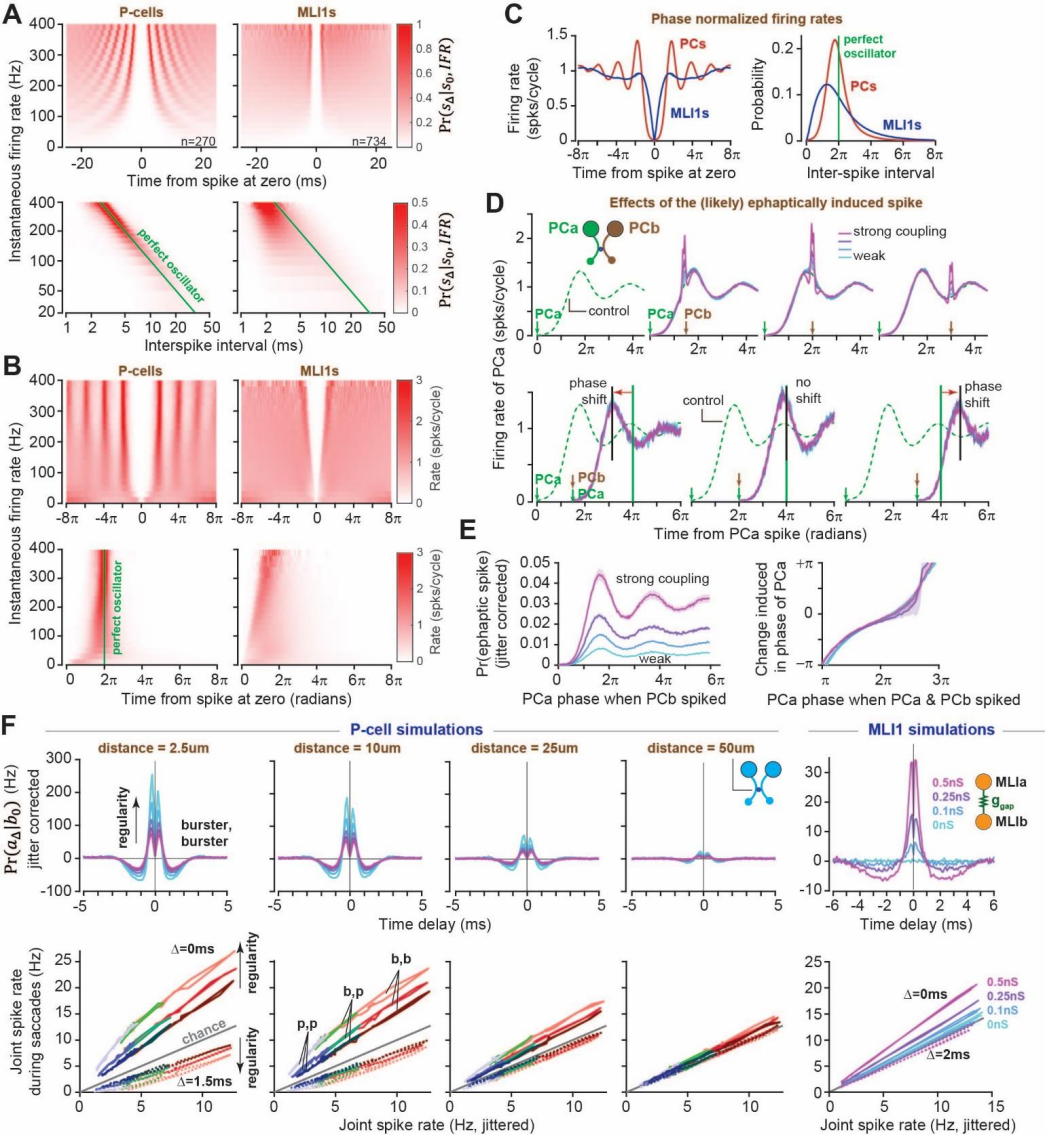
Spike timing of P-cells but not pMLI1s show reliance on an internal clock. Nearby P-cells can reset the phase of this clock through ephaptic coupling. **(A)** Average 3D-autocorrelograms (top) and 3D-isi distribution (bottom) of all P-cells (left) and pMLI1s (right). **(B)** Same as (A) but in phase domain (time normalized by instantaneous firing rate). **(C)** Average auto-correlogram (left) and isi distribution (right) of P-cells and pMLIs in phase domain. **(D)** (Top) Conditional probability of PCa firing at each phase given a neighboring PCb within a clique spiked at a future phase relative to PCa: from left to right: Control (no PCb spike), PCb at 7*π*/5, 2*π*, and 3*π*. (bottom) Probability of PCa firing at each phase given PCa fired again less than 0.5 ms after a PCb spike occurring at some phase Δ relative to PCa (from left to right Δ = 7*π*/5, 2*π*, and 3*π*) grouped by different PCa and PCb ephaptic coupling strength. **(E)** (left) Probability of ephaptic spike (jitter corrected joint spiking of PCa and PCb) given PCb spiked at different phases of PCa grouped by different PCa and PCb ephaptic coupling strength. (right) Change induced in phase of PCa as a function of when the ephaptic spike occurred. **(F)** Joint-jitter plots of simulated P-cell pairs (pair type denoted by color: red for burster-burster pairs, green for burster-pauser pairs, blue for pauser-pauser pairs) with different strengths of ephaptic coupling (decreasing strength from left to right) and regularities (increasing regularity from darker colors to lighter colors) from −50 to 100ms relative to saccade deceleration onset. Simulations for pMLI1 pairs are also shown. Error bars are SEM.

To answer this question, we devised a way to measure the probability of spiking in terms of the phase of the neuron’s imaginary clock. We began by plotting the 3D-autocorrelogram^30^, i.e., the conditional probability of spiking at time *t* + Δ, given that the cell spiked at time *t*, binned by the instantaneous firing rate of that neuron. The results are shown for a sample of individual cells in Fig. S11A and are summarized in Fig. 5A (top row). The curtain-like bands in the P-cells indicate that regardless of firing rate, they tend to spike after a duration inversely proportional to their instantaneous firing rate. In contrast, this relationship was not present in pMLI1s. In the P-cells, the probability peaks became sharper as firing rates increased, indicating greater regularity (Fig. S11B), something that has also been observed for P-cells in the flocculus^31^. Thus, the analogy of an internal clock was appropriate for the P-cells, but not for the pMLI1s.

If we view the P-cells as relying on an internal clock, then we can think of the passage of time between two consecutive spikes in terms of the phase of this clock. We obtained an estimate of the phase by multiplying the time elapsed from each spike by the instantaneous firing rate at the time of that spike (see Methods). This representation of time produced a new way to view the conditional probability, i.e., not in terms of time that had passed from one spike to the next, but in terms of the phase of the clock (Fig. 5B). For the P-cells, but not the pMLI1s, the result was a wave function with peaks at multiples of 2*π*. This wave function maintained its peak position at multiples of 2π regardless of the P-cell’s firing rate, which implied that the P-cells resembled tunable clocks that could run fast or slow. However, regardless of the speed of this clock, i.e., regardless of their instantaneous firing rate, P-cells (but not the pMLI1s) spiked with high probability when their clock reached 2*π* (Fig. 5C).

Now imagine that the clock of a P-cell (PCa) is running and has reached *π*, i.e., half of its cycle. At this time PCb interrupts PCa’s clock by ephaptically generating a spike, thus moving PCa’s phase suddenly to 2*π*. Does this interruption reset the clock of PCa?

To answer this question, for each P-cell within a clique we plotted its normalized firing rate as a function of phase, i.e., its wave function (Fig. 5D, green dashed line). When PCb, a neuron in the same clique as PCa, generated a spike, it increased the probability of spiking in PCa (Fig. 5D, top row). Occasionally, at extremely low latency after PCb spiked (less than 0.5ms), PCa also spiked. These [PCb, PCa] joint spike pairs could result either from random coincidence or from ephaptic induction. To test whether a joint spike could reset PCa’s phase, we grouped P-cell pairs by the strength of their ephaptic coupling (synchrony index, Fig. 2) and examined whether the joint spike acted like a regular spike in resetting the clock. When the joint spike occurred before 2π, the subsequent conditional spiking probability retained the same oscillatory nature, but with a phase shift relative to control, so that the peaks in spiking probability now occurred earlier. This effect was consistent across all levels of ephaptic coupling strength, suggesting that the joint spike reset the phase no differently than spontaneous ones (Fig. 5D, bottom row). When the joint spike occurred after 2π, a phase delay was introduced in the conditional spiking probability, so that the peaks were now later than the peaks in the control. In contrast, if the joint spike occurred near 2π, there was minimal shift in the conditional spiking probability.

We next estimated the probability of an ephaptically induced spike through subtraction of joint spike rates from chance (jittered PCb spikes). P-cells with stronger couplings showed higher probabilities of estimated ephaptic spikes for all phases of PCa phase (Fig. 5E, left). Spikes from a neighboring P-cell were most effective at inducing ephaptic spikes in PCa when the phase of PCa was close to a multiple of 2π (i.e. when PCa was already close to firing), regardless of ephaptic coupling strength (Fig. 5E, left). Additionally, regardless of the strength of their coupling, the change induced in the phase of PCa by the joint spike was roughly a linear function of when that spike occurred (Fig. 5E, right). For example, if this spike occurred before PCa would have normally spiked (before 2π), it moved the next PCa spike to an earlier phase in the cycle, and if it occurred after PCa would have normally spiked (after 2π), it moved the next PCa spike to a later phase in the cycle. To check whether these results were not trivial, we confirmed that pMLIs did not show evidence of phase resetting (Fig. S16).

Thus, the P-cells (but not the pMLI1s) had regular timing in the sense that regardless of their instantaneous firing rates, they tended to spike when the phase of their internal clock was a multiple of 2*π*. When a P-cell induced a potentially ephaptic spike in its neighbor, that spike reset the neighbor’s internal clock. The ephaptic spike had two effects: it produced a near synchronous spike in the two P-cells, and it also reset the phase of the neighboring P-cell, aligning the internal clocks of the two neighbors.

### Ephaptic coupling and clock-like regularity combined to increase synchrony while suppressing asynchrony

While the peak firing rates of P-cells and pMLI1s were comparable (Fig. 3A), synchrony index was significantly greater among the P-cells (Fig. 2F & 2G, t(2900)=21.27, p=1.9×10^−93^). Did regularity combine with electrical coupling to enhance coordination of spike timing among the P-cells?

We used simulations to determine the specific roles that gap junctions, ephaptic coupling, and regularity played in coordinating spike timing among pairs of neurons. We began with an examination of the effects of gap-junctions on the spike timing of pairs of simulated MLI1 neurons. We modeled a pair of conductance-based MLI1 neurons that received parallel fiber inputs via granule cells, which in turn received inputs from 1–5 mossy fibers (Fig. S12). The strength of the gap junction between the two MLI1s was modeled as a conductance term (Fig. S12B). The data for the mossy fibers were from 222 neurons (“state” mossy fibers) recorded during saccades^7^, represented as average firing rates aligned to saccade peak velocity. Excitatory conductance was tuned to reproduce the observed MLI firing rates and AMPA-like temporal dynamics. The gap-junction conductance was systematically varied, and the cross-correlogram (Fig. 5F, right column, top row) and joint-jittered spike probability (Fig. 5F, right column, bottom row) were computed between the two MLI1 neurons, aligned to saccade peak velocity.

The simulations revealed that as gap-junction strength increased, so did the double-peak structure of the cross-correlograms. In contrast, without gap-junctions the simulated MLI1 pairs exhibited only rate correlations, i.e., synchronous spike rates were at chance. Furthermore, as MLI1 firing rates increased, the rate of synchronous spikes (Δ = 0ms) rose above chance while the rate of asynchronous spikes (Δ = 2ms) stayed near chance. Thus, gap-junctions between two simulated MLI1s reproduced the pattern of spike timing that we had recorded in pairs of pMLI1s.

Unlike MLI1s, which exhibited relatively homogeneous firing patterns during saccades, P-cells displayed heterogeneous activity characterized by bursts and pauses. To model P-cell dynamics using a leaky integrate-and-fire (LIF) framework, we adopted a simplified approach that did not explicitly simulate the upstream MLI1 and granule cell circuitry. Instead, we implemented a current-based LIF model that directly controlled the average firing rate of the modeled P-cell so that it reproduced the recorded data in terms of bursting or pausing during saccades (Fig. S13A). This model included the ability to set the regularity in the spike timing of individual P-cells (Fig. S13B), as well as the strength of ephaptic coupling between pairs of P-cells (Fig. S13C).

We simulated two P-cells with different combinations of bursting or pausing activity, generating spike trains and quantified their temporal coordination using cross-correlograms and joint-jitter plots. Focusing on pairs of bursters, we found that increasing ephaptic coupling enhanced spike synchrony at Δ = 0ms while suppressing asynchrony at Δ = 1.5ms (Fig. 5F, effect of distance). Importantly, producing more regular spiking magnified these two features (Fig. 5F, effect of regularity).

Why should more regular spiking magnify the effects of ephaptic coupling? When one neuron spikes, the extracellular voltage changes by a small amount, typically by 0.5 mV at distances of just a few micrometers. Thus, to a first approximation, a spike in one P-cell (PCa) can induce a spike in another P-cell (PCb) if the membrane voltage of PCb is within 0.5 mV of the spiking threshold. We will refer to this voltage region as the ephaptic spiking band. The probability that any randomly selected PCa spike induces a spike in PCb depends on how much time the membrane voltage of PCb spends in this ephaptic spiking band. If PCb is extremely regular, the amount of time spent in the ephaptic spiking band will be roughly proportional to the width of the band (Fig. S17, left). However, if the neuron has irregular spiking, then there will be more noise in the membrane voltage. As a result, whenever it gets close to the ephaptic spiking band, the noise will either push the voltage back down or abruptly push it through the entire band very rapidly. Indeed, as noise increases, the proportion of time spent in the ephaptic spiking band plummets (Fig. S17, right).

Additionally, we noticed that when the simulated P-cells were very regular and ephaptic coupling was very strong (first column of Fig. 5F), the joint-jitter relationship not only deviated further from chance but also became increasingly nonlinear, thereby violating Eq. (1). This nonlinearity can be intuitively understood in the extreme case of very strong ephaptic coupling, where the spike trains in the two P-cells become nearly identical. In this regime, the joint probability of spiking approaches the firing probability of a single neuron, while the jittered probability scales with its square, producing a square-root-like relationship. Fig. 5F also illustrates that in the absence of ephaptic coupling, regardless of spike regularity, the joint probability remains at chance, thus explaining why P-cells in disparate cliques did not exhibit synchrony.

In summary, simulations suggested that the gap-junctions between MLI1s were sufficient to reproduce the temporal coordination observed in our recorded pMLI1s, and ephaptic coupling was sufficient to reproduce the coordination observed in our recorded P-cells. It appeared that the clock-like regularity of spike timing in the individual P-cells combined with ephaptic coupling to increase the rate of synchronous spiking while actively suppressing asynchronous spikes. Our simulations produced a linear joint-jitter relationship that matched Eq. (1) in the range of our empirical data. However, broader exploration across the cerebellar cortex will be necessary to determine whether regions containing more regularly spiking P-cells^32–34^ exhibit nonlinear joint-jitter relationships. Additionally, more work is needed to determine whether the relationship between regularity and synchrony exists for real P-cells, since the simulated model is of course a simplification of the biological reality.

## Discussion

We discovered that spike timing in two different classes of electrically coupled inhibitory neurons, P-cells and pMLI1s, obeyed a mathematical pattern: as the firing rates of individual neurons increased, the rate of joint spikes that were separated by a given time interval rose linearly with respect to chance such that certain temporal intervals were promoted while other intervals were suppressed. For example, the rate of synchronous spikes at 0 ± 0.5ms interval rose disproportionately while the rate of asynchronous spikes at 1.5 ± 0.5ms was suppressed for P-cells and remained at chance for pMLI1s. To understand the purpose of this coordination, we isolated thousands of neuron triplets, each consisting of a pair of pMLI1s that interacted with a target P-cell. When the upstream pair spiked synchronously, the downstream P-cell experienced a doubling of the inhibition, then a doubling of the post-inhibitory rebound. However, when the upstream spikes were 2–3ms apart, the effect was mutual interference. Thus, electrical coupling among pairs of inhibitory neurons enhanced constructive superposition upon their mutual target while suppressing destructive competition.

Remarkably, spike timing in the individual P-cells exhibited a clock-like pattern that was not present in the pMLIs. That is, unlike the MLI1s, each P-cell behaved as an oscillator in which the probability of spiking varied with the phase of its internal clock. When a P-cell ephaptically induced a spike in its neighboring P-cell, that spike reset the phase of the neighboring clock so that the two clock phases became aligned. Thus, a single ephaptic interaction altered the spike timing probability of a P-cell for multiple spikes. Simulations showed that this clock-like conformity in spike production magnified the effects of ephaptic coupling. That is, regularity in spike timing combined with ephaptic coupling to increase the rate of synchronous spikes.

### Spike coordination was present only in neurons that belonged to the same clique

Clustering the neurons into small, connected networks, i.e., cliques, was essential for unmasking the organization of spikes^7^. Without it, spike timing in neuron pairs remained near chance. Because our electrodes were driven perpendicular to the layers, we inferred that each clique was confined to a single P-cell layer, but we could not measure the span of the clique within that layer. For example, it is possible that within a layer each clique had a width that ran along the parallel fibers but perhaps not perpendicular to it^35^.

The interactions within pMLI1 pairs were consistent with gap-junctions^3,36^, which connected neighboring MLIs into small networks in which spikes tended to synchronize^4,6^, especially following the onset of a movement^37^. Our simulations of gap-junctions in the MLIs and ephaptic coupling in the P-cells reproduced the linear relationship between joint and jittered rates (Eq. 1), revealing that as the strength of electrical coupling increased, so did the rate of synchronous spikes, but with little change in the firing rates of the individual MLIs. Thus, electrical coupling did not produce additional spikes, but rather served as a mechanism to coordinate the timing of those spikes, consistent with the results in the cerebral cortex^38^.

### Joint-jitter plots resolved controversies in measuring coordination of spike timing

One method for asking whether the rate of synchronous events is different than chance is to measure the ratio of joint probability to the independent probabilities, termed the synchrony index^39^. However, this approach faces the limitation that as the firing rates decline to near zero, for example in P-cell pausers, the ratio becomes noisy or undefined^40^. To remedy this, Herzfeld et al.^26^ suggested measuring the difference between the rate of synchronous events and the independent probabilities, termed covariance. However, in principle the joint rate can be an arbitrary function of the states of the two neurons, making it unclear whether synchrony index or covariance is the proper measure.

Here, we quantified the empirical relationship between the joint probability and the independent probabilities for each neuron pair over hours of recording as the subject was engaged in the task. These joint-jitter plots unmasked a striking pattern: the joint rates at various spike temporal intervals varied roughly linearly with respect to the jittered rates. That is, regardless of whether cells increased or decreased their rates, synchrony index remained approximately constant, but not covariance. Our new method of visualizing the data revealed that as the firing rates of individual neurons increased, the temporal interval of spikes between two neurons was not random but coordinated to promote specific intervals while suppressing others. The intervals that were promoted were those that produced downstream superposition, while the intervals that were suppressed were those that caused interference.

### Ephaptic coupling and clock-like regularity combined to increase synchrony

What was the functional significance of the regularity in P-cell spike timing? Payne et al.^31^ investigated this question by controlling the timing of spike production in groups of P-cells via optogenetic stimulation. They demonstrated that each pulse of stimulation moved the eyes by a small amount, but that effect was not dependent on whether the stimulation timing was regular or irregular. Here, we considered the fact that neighboring P-cells exhibited diverse firing rates during saccades, some bursting and others pausing. However, each P-cell maintained a clock-like regularity in its timing. In our simulations, spike time regularity enhanced synchrony, generating a better alignment of spikes in pairs of P-cells. Thus, regularity in spike timing acted in concert with ephaptic coupling, elevating the rate of synchronous spikes above chance while suppressing the rate of asynchronous spikes below chance.

### A rationale for coordination of P-cell spike timing

It is exceedingly difficult to simultaneously record from a pair of P-cells that project onto the same nucleus neuron, particularly in an awake, behaving animal. As a result, we do not know whether spike coordination among pairs of P-cells matters from the perspective of their shared downstream nucleus neuron. However, our data from pairs of MLIs that jointly inhibited a target P-cell provided important clues.

The pMLI1 data demonstrated that whereas synchronous spikes produced superposition on the target P-cell, spikes that were but a few milliseconds apart were destructive. Roughly 50 MLI1s converge onto the pinceau of a single P-cell^41^, and around 50 P-cells converge onto a single nucleus neuron^42^. We found that like the pMLI1s, the P-cell pairs exhibited greater than chance probability of jointly spiking within 1ms of each other, but less than chance probability of spiking within 2ms. The inhibitory post-synaptic current produced by a single P-cell spike in the nucleus neuron has a time constant of 2.5ms^42^. This extremely fast time constant implies that, like the effect of the MLIs on the P-cells, only synchronous P-cell spikes can induce superposition of their individual effects in the target nucleus neuron. The fact that 2ms spike intervals were suppressed among P-cell pairs predicts that like pMLI1s, this interval should be avoided because it causes destructive interference in the nucleus neuron.

In the pausing P-cells the firing rates declined during saccades, while in the bursting P-cells the firing rates increased. Thus, only in the bursting pairs the number of synchronous spikes increased. Did pausing and bursting P-cells play different roles in driving the nucleus neurons? Gauck and Jaeger^43^ found that a transient reduction in the firing rates of the inhibitory input to a nucleus neuron resulted in spiking in the nucleus, as did production of synchronized input to the nucleus. However, an increase in the inhibitory input had little effect. Nashef et al.^44^ confirmed this, demonstrated that nucleus neurons responded strongly when inhibition was reduced while inputs became more synchronized. This implies that the cerebellar cortex combines two different mechanisms to drive the activity of the nucleus neurons: reduced inhibition via pausing P-cells, and increased synchrony via bursting P-cells.

Whereas synchronous spiking among P-cells has been shown to entrain downstream nucleus neurons^42,43,45^, a recent work found that synchronous pausing, i.e., not spiking for relatively long periods, also has a causal effect on nucleus firing^46^. Indeed, there is evidence that synchronous pausing is also regulated among pairs of P-cells^47–50^. In the zebra fish cerebellar nucleus, the activities of the nucleus-like neurons cannot be reproduced via mean rates of inhibitory and excitatory inputs, because on average, mean inhibition far exceeds excitation. However, firing rates in the nucleus depend on precise timing of the inhibitory and excitatory inputs, allowing for gaps in inhibition to bring the neuron to threshold^46^.

Thus, both the rate and timing of P-cell spikes, including millisecond-scale synchrony and coordinated pauses, appear critical for shaping the output of cerebellar nucleus neurons.

### The role of MLIs in coordinating spike timing in pairs of P-cells

Parallel fibers provide excitatory inputs to both the P-cells and the MLIs, resulting in a feedforward inhibitory network in which inhibition can rapidly quench the excitability window^51^. The MLIs employ the pinceau to generate possibly the fastest inhibition in the entire brain^18^. The electrical coupling between the MLIs appears to make them particularly responsive to synchronous events in the parallel fibers^52^, raising the possibility that the role of the feedforward inhibitory network is to make P-cells respond mainly to coincident events in their parallel fibers. In addition, it is possible that a component of spike coordination among pairs of P-cells may be due to a network wide organization that involves the MLIs^53^. However, without the GABA induced inhibition from the MLIs, P-cell spike timing becomes more regular^10,12^. Our simulations show that the regularity in P-cell spike timing increases the synchrony between pairs of P-cells. Thus, on the one hand MLI1s may endow P-cells with coincidence detection in their parallel fiber inputs, while on the other hand countering the regularity of P-cells and thus reducing pair-wise synchronization.

### Spatial organization of temporal coordination

Although the joint firing rates in pairs of P-cells were an order of magnitude smaller than the individual firing rates, the convergence of roughly 50 P-cells onto each downstream nucleus neuron could make their combined effects comparable in magnitude at the network level. Due to our recording limitations, the spatial organization of the P-cells and their connectivity—whether they coordinate along a line or within a grid—remains unknown. However, previous work suggests that these interactions are not random but spatially structured^5,54^. The connectivity matrix of P-cells (Fig. 1E) varies across cerebellar regions^55^, and such structured connectivity, together with their downstream projections, can shape both the activity of target neurons and the spiking patterns within cliques themselves (Fig. 5). Further theoretical work and recordings that span larger areas of the cerebellar cortex using multi-shank probes will be essential to reveal the emergent network properties arising from these couplings.

### Cerebellar disease typically accompanies disruption of spike timing, not the average firing rates

Genetic mutations that disrupt function of calcium channels generally do not affect average firing rates of P-cells during a visuomotor task, but instead alter their clock-like regularity, which coincides with behavioral symptoms^56^. Similarly, in Duchenne muscular dystrophy, P-cell average firing rates remain stable while regularity is altered^57^. In episodic ataxia type-2, P-cells exhibit reduced spike time regularity^58^, and the drug 4-AP improves the symptoms while restoring P-cell regularity^59^. However, a recent study showed that regularity does not encode any information beyond what is already present in the neural firing rates, leaving the question of how regularity contributes to behavior unanswered^31^. Here, we found that regularity may combine with ephaptic coupling to increase temporal coordination of spikes in P-cell pairs. We suggest that in diseases in which P-cell pairs exhibit reduced temporal coordination, an increase in the firing rates during behavior will result in greater destructive interference and reduced constructive superposition in the downstream nucleus neurons, impairing the ability of the cerebellar cortex to transmit information to its downstream target.

## Supplementary Material

This is a list of supplementary files associated with this preprint. Click to download.

- supplementarymaterials.docx

## Main findings

- Spike timing among neighboring P-cells and MLIs exhibited a mathematical regularity.
- As firing rates increased, the rate of 1ms spike intervals grew disproportionately while 2–4ms intervals remained at or below chance.
- The 1ms intervals between pairs of MLIs produced superposition on the downstream P-cell, whereas 2–4ms intervals produced interference.
- Individual P-cell spike timing exhibited reliance on an internal clock. When a P-cell generated a spike, ephaptic coupling reset the internal clock of its neighboring P-cell, transiently aligning the clocks of the two cells.
- Electrical coupling among neighboring inhibitory neurons produced constructive superposition of their individual inhibitions on their downstream neurons while simultaneously reducing spiking events that resulted in counterproductive competition.
